# Retrospective evaluation of exposure index (EI) values from plain radiographs reveals important considerations for quality improvement

**DOI:** 10.1002/jmrs.25

**Published:** 2013-11-20

**Authors:** Ursula Mothiram, Patrick C Brennan, John Robinson, Sarah J Lewis, Bernadette Moran

**Affiliations:** 1 Medical Imaging Optimisation and Perception Group (MIOPeG), Discipline of Medical Radiation Sciences, Faculty of Health Science, University of SydneySydney, Australia; 2 Department of Clinical Medicine, Trinity College DublinDublin, Ireland

**Keywords:** Computed radiography (CR), digital radiography (DR), exposure index (EI), plain radiographs, quality improvement

## Abstract

**Introduction:**

Following X-ray exposure, radiographers receive immediate feedback on detector exposure in the form of the exposure index (EI).

**Purpose:**

To identify whether radiographers are meeting manufacturer-recommended EI (MREI) ranges for routine chest, abdomen and pelvis X-ray examinations under a variety of conditions and to examine factors affecting the EI.

**Methods:**

Data on 5000 adult X-ray examinations including the following variables were collected: examination parameters, EI values, patient gender, date of birth, date and time of examination, grid usage and the presence of implant or prosthesis. Descriptive statistics were used to summarize each data set and the Mann–Whitney *U* test was used to determine significant differences, with *P* < 0.05 indicating significance for all tests.

**Results:**

Most examinations demonstrated EI values that were outside the MREI ranges, with significantly higher median EI values recorded for female patient radiographs than those for male patients for all manufacturers, indicating higher detector exposures for all units except for Philips digital radiography (DR), where increased EI values indicate lower exposure (*P =* 0.01). Median EI values for out of hours radiography were also significantly higher compared with normal working hours for all technologies (*P* ≤ 0.02). Significantly higher median EI values were demonstrated for Philips DR chest X-rays without as compared to those with the employment of a grid (*P* = 0.03), while significantly lower median EI values were recorded for Carestream Health computed radiography (CR) chest X-rays when an implant or prosthesis was present (*P* = 0.02).

**Conclusions:**

Non-adherence to MREIs has been demonstrated with EI value discrepancies being dependent on patient gender, time/day of exposure, grid usage and the presence of an implant or prosthesis. Retrospective evaluation of EI databases is a valuable tool to assess the need of quality improvement in routine DR.

## Introduction

Digital radiography (DR) was first introduced almost 30 years ago and it is now considered standard technology in most radiology centres.^1^ The transition to digital technologies has brought about a much wider dynamic range of radiographic exposure parameters than that of conventional film screen.^1^,^2^ This means that a diagnostic image can be generated over a much greater range of entrance doses,^1^ without an adverse effect on image quality.^3^–^5^ There is, however, a need to ensure that with an increased dynamic range, patient radiation doses are not excessive; therefore, an indication of exposure is required. With the current digital radiographic technology, this comes in the form of an exposure indicator that specifically offers feedback on the radiation level reaching the imaging detector.^2^,^6^ However, it is important to note that the exposure index (EI) is not an actual measure of patient dose.^7^

Currently, there are a variety of manufacturer-dependent EIs, which presents the opportunity for user confusion. To counter this, an international standardized EI has been developed simultaneously by the International Electrotechnical Commission (IEC) and the American Association of Physicists in Medicine (AAPM) in conjunction with the DR system manufacturers.^8^ The standardized EI has been designed to generate a linear relationship between the detector exposure and index value.^8^,^9^

This study demonstrates the usefulness of careful examination of EI data, indicating for example the influence of patient type, technology and radiographic practice on EI values. It examines the EIs recorded for routine adult X-ray examinations of the chest, abdomen and pelvis to assess adherence to manufacturers' EI recommendations and it also explores how the EI values are affected by parameters such as patient gender, time or date of exposure, the use of secondary radiation grid and the presence of implant or prosthesis.

## Materials and Methods

### Equipment

The study was performed in one of the largest teaching hospitals in Ireland that operates in a full picture archiving and communication system (PACS) environment. The authors have chosen to keep the hospital name anonymous to ensure the anonymity of the radiographers working there. Chest, abdomen and pelvis examination details were extracted following image acquisition using Siemens Medical VX and MX Axiom Aristos DR, a Philips DR DigitalDiagnost system version 1.4 and a Carestream Health Direct View CR500 computed radiography (CR). The Philips and Siemens DR systems utilize flat panel detector technologies.

Details of the grids used are (in the order grid ratio [*R*], line frequency [*N*] and focal distance [*F*]):

- Siemens: *R* = 15:1, *N* = 80 lines/cm, *F* = 115 cm; *R* = 15:1, *N* = 80 lines/cm, *F* = 180 cm
- Philips: *R* = 8:1, *N* = 36 lines/cm, *F* = 110 cm; *R* = 8:1, *N* = 36 lines/cm, *F* = 140 cm

The allocation of specific examinations to specific technologies is demonstrated in Tables 2–4. All the pieces of equipment included in this study have been calibrated according to manufacturers' recommendations. In addition, for both Philips and Siemens equipment, the radiographers are also periodically prompted to perform a system calibration by the system software. The Carestream Health CR plates are calibrated at 6-monthly intervals during scheduled maintenance. In addition, the CR readers are calibrated at 6-monthly intervals and matched. The systems are also closely monitored by hospital medical physics staff.

**Table 1:** The manufacturer-recommended exposure index value ranges.

Manufacturer/model	MREI range	Exposure indicator
Siemens Medical VX and MX Axiom Aristos DR (Erlangen, Germany)	150–400 (Schramm H, 2012, personal communication)	EXI^6^
Philips DR DigitalDiagnost system version 1.4 (Hamburg, Germany)	250–630 (Neitzel U, 2012, personal communication)	EI^6^
Carestream Health Direct View CR500 CR (Rochester, New York)	1700–1900^12^,^13^	EI^6^

DR, digital radiography; CR, computed radiography; MREI, manufacturer-recommended exposure index; EXI, exposure index; EI, exposure index. [Correction added on 27 November 2013, after first online publication: In the first row of Table 1, ‘EXI’ has been corrected from ‘EI’, and the corresponding legend ‘EXI, exposure index;’ has been inserted.]

**Table 2:** Descriptive statistics of exposure index value distributions for the manufacturer/examinations included in this study (for manufacturer-recommended exposure index value ranges see Table 1).

					Exposure	
					
Manufacturer/examination	Number (*n*)	Median	First quartile (*Q*_1_)	Third quartile (*Q*_3_)	% Over	% Under
Siemens DR						
Chest posterior–anterior	2453	228	195	267	1	6
Chest lateral	107	319	278	360	13	1
Chest supine	15	226	194	289	0	0
Chest lateral decubitus	2	193	142	243	0	50
Chest anterior–posterior	4	249	146	270	0	25
Abdomen	377	233	205	256	0	1
Pelvis	239	467	369	581	67	0
Philips DR						
Chest posterior–anterior	213	400	320	400	0	1
Chest lateral	15	400	320	400	0	0
Chest anterior–posterior	9	400	250	630	11	11
Abdomen	26	630	630	630	0	0
Pelvis	3	630	500	630	0	0
Carestream Health CR						
Mobile chests	1261	2040	1930	2170	79	3
Departmental chests	273	2020	1880	2130	72	4
Abdomen	1	1400	1400	1400	0	100
Pelvis	2	1650	1510	1790	0	50
Total	5000				28	4

DR, digital radiography; CR, computed radiography.

**Table 3:** Exposure index values for each patient gender and examination type included in this study (for manufacturer-recommended exposure index value ranges see Table 1).

						Exposure		
							
Manufacturer/examination	Gender	Number (*n*)	Median	First quartile (*Q*_1_)	Third quartile (*Q*_*3*_)	% Over	% Under	*P*-value
Siemens DR								
**Chest posterior–anterior**	**F**	**1207**	**239**	**209**	**275**	**1**	**3**	**<0.0001**
	**M**	**1246**	**216**	**185**	**255**	**1**	**8**	
**Chest lateral**	**F**	**48**	**332**	**285**	**409**	**29**	**0**	**0.04**
	**M**	**59**	**313**	**268**	**343**	**2**	**2**	
**Chest supine**	**F**	**8**	**263**	**218**	**306**	**0**	**0**	**0.03**
	**M**	**7**	**201**	**180**	**227**	**0**	**0**	
Chest lateral decubitus	F	2	193	142	243	0	50	nf
	M	0				0	0	
Chest anterior–posterior	F	2	269	267	271	0	0	nf
	M	2	174	118	230	0	50	
**Abdomen**	**F**	**204**	**227**	**199**	**252**	**0**	**2**	**0.001**
	**M**	**173**	**240**	**212**	**265**	**0**	**0**	
Pelvis	F	157	479	384	596	70	0	ns
	M	82	447	357	526	62	0	
Philips DR								
**Chest posterior–anterior**	**F**	**90**	**400**	**400**	**400**	**0**	**0**	**0.01**
	**M**	**123**	**400**	**320**	**400**	**0**	**1**	
Chest lateral	F	3	400	400	400	0	0	ns
	M	12	400	320	475	0	0	
Chest anterior–posterior	F	5	320	225	515	20	0	ns
	M	4	630	345	758	0	25	
Abdomen	F	17	630	630	630	0	0	ns
	M	9	630	630	630	0	0	
Pelvis	F	2	565	500	630	0	0	nf
	M	1	630	630	630	0	0	
Carestream Health CR								
Mobile chests	F	483	2060	1930	2190	81	3	ns
	M	778	2030	1920	2160	78	3	
**Departmental chests**	**F**	**148**	**2055**	**1910**	**2160**	**77**	**3**	**0.001**
	**M**	**125**	**1970**	**1855**	**2080**	**66**	**5**	
Abdomen	F	0				0	0	nf
	M	1	1400	1400	1400	0	100	
Pelvis	F	2	1650	1510	1790	0	50	nf
	M	0				0	0	
Total		5000						

Rows with values in bold indicate significant differences in median exposure index values (*P* < 0.05), where medians were equal the mean was used to determine significance. M, male; F, female; nf, numbers too few; ns, not significant; DR, digital radiography; CR, computed radiography.

**Table 4:** Exposure index values for in hours and out of hours examinations (for manufacturer-recommended exposure index value ranges see Table 1).

						Exposure		
							
Manufacturer/examination	In hours (I)/out of hours (O)	Number (*n*)	Median	First quartile (*Q*_1_)	Third quartile (*Q*_3_)	% Over	% Under	*P*-value
Siemens DR								
**Chest**	**I**	**1553**	**224**	**192**	**258**	**1**	**6**	**<0.0001**
	**O**	**1028**	**245**	**204**	**287**	**2**	**5**	
**Abdomen**	**I**	**210**	**224**	**194**	**251**	**0**	**2**	**<0.0001**
	**O**	**167**	**241**	**215**	**261**	**0**	**0**	
Pelvis	I	194	469	376	568	69	0	ns
	O	45	428	343	615	62	0	
Philips DR								
**Chest**	**I**	**52**	**400**	**320**	**400**	**0**	**0**	**0.01**
	**O**	**185**	**400**	**400**	**400**	**1**	**1**	
Abdomen	I	7	630	630	630	0	0	ns
	O	19	630	630	630	0	0	
Pelvis	I	2	630	630	630	0	0	nf
	O	1	500	500	500	0	0	
Carestream Health CR								
**Mobile chests**	**I**	**596**	**2020**	**1910**	**2150**	**77**	**3**	**0.0005**
	**O**	**665**	**2060**	**1940**	**2190**	**82**	**3**	
**Departmental chests**	**I**	**139**	**1970**	**1840**	**2120**	**66**	**5**	**0.02**
	**O**	**134**	**2050**	**1910**	**2140**	**78**	**3**	
Abdomen	I	1	1400	1400	1400	0	100	nf
	O	0				0	0	
Pelvis	I	1	1510	1510	1510	0	100	nf
	O	1	1790	1790	1790	0	0	
Total		5000						

Rows with values in bold indicate significant differences in median exposure index values (*P* < 0.05), where medians were equal the mean was used to determine significance. nf, numbers too few; ns, not significant; DR, digital radiography; CR, computed radiography.

### Data collection

The study was conducted from November 2010 to August 2011. EIs were retrospectively recorded on a total of 5000 routine examinations performed over a 35-day period between October and November 2010 in a busy teaching hospital. Recorded information consists of chest (*n* = 4352), abdomen (*n* = 404) and pelvis (*n* = 244) X-ray examinations. The examination details for each patient were retrieved from the hospital PACS. The specific examination types are shown in Tables 2–4. Data were gathered on adult male (2622) and female (2378) patients and individuals' details were anonymized to protect the privacy of patients. Ethics approval was granted by the Human Research Ethics Committee of the University of Sydney and the Ethics Committee and Risk and Legal departments within the hospital in which the study was based.

In addition to the EI, the following details were recorded for each examination: patient gender, time and date of exposure, and the use of a secondary radiation grid. When an image demonstrated the presence of an implant or prosthesis, this was also recorded as the literature has indicated this can affect EI values.^10^ Only initial presentation examinations were recorded as additional specialized projections may not be indicative of normal practice. The data on the date and the time of taking the radiographic exposures were used to categorize the radiographs into two: those taken ‘in hours’ and ‘out of hours’. Normal working hours (or in hours) were established as 9 a.m. to 5 p.m. Monday to Friday and out of hours included public holidays, weekends and from 5 p.m. to 9 a.m. Monday to Friday.

### Data analysis

The data were analysed in several ways. GraphPad Prism 5 software (La Jolla, CA) was used to perform statistical analysis. Descriptive statistics were used to summarize the characteristics of each data set relating to specific manufacturer and examination type.^11^ Exposure indices for each examination were compared to manufacturer-recommended EIs (MREIs) for specific equipment (see Table 1) and the percentage of values not adhering to recommendations was calculated.

As the conditions for normality were not met the Mann–Whitney *U* test was then used to determine significant differences between EI values, for each examination type, for the following paired comparisons:

- male versus female patients;
- in hours versus out of hours;
- use versus non-use of secondary radiation grid (this was only possible for Philips DR chest examinations);
- presence versus absence of an implant or prosthesis (examples of implants and prostheses included pacemakers, orthopaedic joint replacements, pins and plates).

A *P*-value of <0.05 was used to describe significant differences.

## Results

Readers are reminded that for the Siemens and Carestream technologies higher EI values indicate a higher radiation exposure compared to lower EIs, whereas the reverse is relevant for the Philips equipment, where higher EI values indicate lower radiation exposure. MREI value ranges for Philips and Siemens DR systems are based on them operating at a sensitivity of 400.

### Descriptive statistics

EI data relating to the examinations included in this study are outlined in Table 2. Most examinations demonstrated that EIs for at least some exposures fell outside the MREI, the exceptions being the following examinations: Siemens supine chest; Philips lateral chest; Philips abdomen; Philips pelvis (see Table 2). Overall 28% of examinations demonstrated EI values above and 4% below the MREI: here, Philips values less than 250 were included in the overexposure category and values greater than 630 were included in the underexposure category.

### Statistically significant results

#### Patient gender

EI variations were evident between the patient genders (see Table 3), with female patients recording significantly higher median EI values than male patients for all the manufacturers for the following examinations: Siemens: chest posterior–anterior (PA) (*P* < 0.0001), lateral (*P* = 0.04) and supine examinations (*P* = 0.03); Carestream Health technology departmental chests (*P* = 0.001); Philips DR PA chest examinations (*P* = 0.01). Conversely male patients recorded higher values than female patients for abdomen examinations (*P* = 0.001) with the Siemens technology.

#### In hours and out of hours

Median EI values out of hours compared to within hours were significantly higher for all technologies: Siemens: chest (*P* < 0.0001) and abdomen examinations (*P* < 0.0001); Carestream: mobile (*P* = 0.0005) and departmental (*P* = 0.02) chest examinations; Philips DR chest X-ray examinations (*P* = 0.01) (see Table 4).

#### Secondary radiation grid

Significantly higher median EI values were demonstrated for Philips DR chest X-rays without grid use (*P* = 0.03) compared to when a grid was employed (see Table 5).

**Table 5:** Exposure index values for Philips digital radiography chest X-rays and grid usage (for manufacturer-recommended exposure index value ranges see Table 1).

					Exposure		
						
Philips chest DR	Number (*n*)	Median	First quartile (*Q*_1_)	Third quartile (*Q*_3_)	% Over	% Under	*P*-value
**Grid in**	**225**	**400**	**320**	**400**	**0**	**0.4**	**0.03**
**Grid out**	**12**	**450**	**288**	**630**	**8.3**	**8.3**	
Total	237						

Rows with values in bold indicate significant differences in median exposure index values (*P* < 0.05). DR, digital radiography.

#### Presence of implant or prosthesis

Lower median EI values were identified with Carestream chest X-rays when an implant or prosthesis was present compared to when an implant or prosthesis was absent (*P* = 0.02).

## Discussion

The purpose of this study was to identify whether radiographers are meeting the MREI ranges for routine chest, abdomen and pelvis examinations and to examine if patient gender, time and day of exposure, use of secondary radiation grid and presence of an implant or prosthesis impacted significantly upon EI values.

Most examinations demonstrated EI values that were outside the MREI ranges. Carestream Health CR mobile and departmental chest X-rays demonstrated the highest overexposure rates of 79% and 72%, respectively, followed by Siemens DR pelvis X-ray examinations, with an overexposure rate of 67%. This apparent overexposure is largely in agreement with the literature, which indicates that transition from conventional film screen radiography to CR or DR can result in an increase in radiation dose to the patient.^5^,^6^ The lowest underexposure rates were demonstrated for Carestream Health CR abdomen and pelvis X-rays and Siemens DR lateral decubitus chest X-rays; however, due to the small sample sizes in these three categories these results should be viewed with caution.

The high rate of overexposures for Carestream Health CR chest X-rays is a concern as it is one of the most frequently performed radiographic examinations,^14^ particularly because clinically acceptable images at EIs lower than the MREI have been shown previously.^13^,^15^ The overexposures attributed to Carestream Health CR mobile chest X-rays may be linked to the complexities associated with mobile chest X-rays where patient position, X-ray source image receptor distances (SID) and exposures are much more variable than in the departmental situation.^16^ There may also be increased pressure on radiographers not wanting to have to repeat the examination due to low-dose-related image mottle so that ward staff exposures and inconvenience are minimized. The recording of previous examination exposure factors with mobile chest radiography has been suggested to assist in reducing patients' radiation doses with film screen technologies.^17^ Here, a clear link between shorter focus to skin distances (FSD) and higher entrance skin doses (ESD) was demonstrated, however, when exposure factors were optimized for different FSDs and when FSDs were measured for each examination dose reductions were achieved.^17^ The potential to lower EIs through optimizing exposures for various FSDs using CR should be investigated. However, more difficult to explain is the excessive exposures evident with the department-based exposures and this requires further investigation. It is important to note that the CR examinations included in this study were not performed in conjunction with an automatic exposure control (AEC).

Although observing non-compliant exposures is a useful quality assurance task, it is probably of greater value to identify factors relating to EI variations and in this study we identified four key agents – patient gender, time/date of exposure, grid usage and the presence of an implant or prosthesis. In terms of patient gender, for most examinations irrespective of the manufacturer, radiographs on female patients demonstrated higher EI values (higher exposures) when compared with male patients. For the Siemens and Carestream technology, this was statistically significant in four instances for female patients compared with only one example demonstrating significantly higher values with male patients. There is no clear justification for this higher exposure pattern in female patients. A previous study has suggested that inadequate exposure charts for female patients could result in higher EI values and recommended optimization of exposure charts to reduce detector dose.^18^ Exposure chart optimization could counteract this gender bias. It has be noted in the literature that variations in male and female doses are relevant for the monitoring of AEC systems: the smaller the variations in mean doses detected the more sensitive the AEC is to changes in the attenuation characteristics of different patients groups.^19^ Although referring to actual radiation dose, this would be worth investigating, as AEC performance could be a factor in the higher female EI values demonstrated in this study using DR. In this study, all the Siemens DR examinations were performed using the AEC. It should be noted that while chest PA examinations with the Philips technology also exhibited higher EI values for female patients compared with male patients, this actually indicates a lower exposure.

Out of hours exposures were also associated with significantly higher EIs for a range of examinations, which has been previously reported and may be linked to reduced on-call staff numbers coupled with a strong reluctance among radiographers to have to repeat an exposure,^15^ when working a busy shift out of hours. The higher EIs could be related to staffing and the level of experience of radiographers performing shift work. Whatever the reason, this is an unacceptable inconsistency that needs to be addressed and highlights the importance of regular monitoring of EI values within departments to highlight when excessive out of hours exposures take place.

A significantly higher median EI value (lower exposure) was demonstrated for Philips DR chest X-rays without a grid. It may be argued that with non-usage of a grid, the exposure levels would be less, but this ignores the fact that the EI is based on pixel values, so these should remain relatively constant regardless of whether a grid has been used. With the DigitalDiagnost system the automatic exposure device (AED) can remain in position when the grid is removed, thus allowing for phototimed exposures to be made with grid in or out.^20^ If AEDs are being used, and because these are positioned on the detector side of the grid,^21^ proper calibration and operation need to be confirmed.^22^ It is also possible that radiographers were using adjustments when using the AEC, which could account for the EI values seen here. Variations in the SIDs applied in conjunction with grid usage in this instance could also have had a bearing on the EIs recorded here.

The reduction in EI values with Carestream Health CR chest examinations in the presence of an implant or prostheses is also notable and highlights that radiographers need to understand the basis on which EI values are calculated before judgements can be made on exposures. In this circumstance, it is possible that the lower EI values reflect the fact that if the prosthesis was within the clinical region of interest from which average pixel values were calculated. Naturally, the reduction in X-rays reaching the detector due to the prosthesis would reflect in lower pixel values and thus lower EIs, and if so, this clearly has little to do with improper technique.

The purpose of this study was to retrospectively analyse EI values and it does not set out to compare the different technologies or manufacturers included in the study nor does it attempt to make a reference to patient dose. As this was a retrospective study it was not possible to analyse patient's size and body part thickness, which is a limitation of this study. Data on these variables could provide useful information relating to the EI gender variations demonstrated here, as male patients and female patients have variation in tissue distribution.

## Conclusion

In summary, this study demonstrates a series of EI values that do not adhere to recommended values. However, to identify which exposures are inappropriate requires that manufacturer levels of EIs be optimized. However, it can be stated with confidence that the variations associated with patient gender, the time or date of exposure, and the use or non-use of a secondary radiation grid are very difficult to justify and corrective strategies should be explored. This study highlights the value of auditing EIs to assist with image quality improvement and ultimately to aid in patient dose reduction.
